# Competencia en Práctica Basada en la Evidencia y factores asociados en las enfermeras de Atención Primaria en España

**DOI:** 10.1016/j.aprim.2021.102050

**Published:** 2021-04-20

**Authors:** Serafín Fernández-Salazar, Antonio Jesús Ramos-Morcillo, César Leal-Costa, Jessica García-González, Solanger Hernández-Méndez, María Ruzafa-Martínez

**Affiliations:** a Estrategia de Cuidados de Andalucía, Servicio Andaluz de Salud, AGS Nordeste de Jaén, Úbeda, Jaén, España; b Departamento de Enfermería, Facultad de Enfermería, Campus de Espinardo, Universidad de Murcia, Murcia, España; c Departamento de Enfermería, Facultad de Ciencias Sociosanitarias, Campus de Lorca, Universidad de Murcia, Lorca, Murcia, España; d Hospital Rafael Méndez, Área III de Salud, Servicio Murciano de Salud, Lorca, Murcia, España

**Keywords:** Práctica Basada en la Evidencia, Competencia, Enfermería, Atención Primaria, Enfermeras comunitarias, EBP-COQ Prof©, Evidence-Based Practice, Competency, Nurse, Primary Health Care, Community nurses, EBP-COQ Prof©

## Abstract

**Objetivo:**

Conocer el nivel de competencia en Práctica Basada en la Evidencia (PBE) de las enfermeras de Atención Primaria (AP) en España y analizar los factores asociados.

**Diseño:**

Estudio transversal de carácter nacional, realizado en enero-marzo de 2020.

**Emplazamiento:**

AP en España.

**Participantes:**

Setecientas ochenta enfermeras de AP en activo en el Sistema Nacional de Salud con experiencia profesional mínima de un año.

**Mediciones principales:**

1) Variables sociodemográficas, profesionales y de acceso a información científica, y 2) variable de resultado: competencia en PBE (actitud, conocimientos, habilidades y utilización), evaluada mediante el cuestionario EBP-COQ Prof©. Se realizaron análisis bivariados y multivariados mediante regresión lineal.

**Resultados:**

La puntuación media en el nivel de competencia en PBE de las enfermeras de AP fue de 131,5 (desviación típica [DT] 17,0). Por dimensiones: actitud 36,8 (DT 3,6); conocimientos 38,2 (DT 8,9); habilidades 23,0 (DT 3,5) y utilización 33,3 (DT 6,1). Leer más de 3 artículos en el último mes es la variable que tiene más influencia sobre todas las dimensiones del EBP-COQ Prof©, seguida de la formación en PBE (más de 150 h) y la tutorización de alumnos de Enfermería. El nivel educativo (máster, especialista y doctorado) se asocia con las dimensiones conocimientos y habilidades, mientras que trabajar en un centro BPSO® se asocia con la utilización de la PBE.

**Conclusiones:**

Estos hallazgos pueden orientar a los gestores en los servicios de AP a planificar estrategias que mejoren el nivel de competencia en PBE de las enfermeras, dirigidas principalmente a lograr una aplicación real en la práctica clínica. No obstante, se hace necesario considerar el posible impacto del sesgo de selección en los resultados.

## Abstract (en)

**Objective:**

To describe the Evidence-Based Practice (EBP) competency level in Primary Care (PC) nurses in Spain and to determine the associated factors.

**Design:**

Cross-sectional, national survey design, carried out between January and March 2020.

**Setting:**

PC in Spain.

**Participants:**

Seven hundred eighty PC active nurses in the National Health Service with at least one year of professional experience.

**Main measurements:**

(1) Sociodemographic, professional and access to scientific information variables; (2) outcome variable: EBP competency (attitude, knowledge, skills and utilization) assessed through the EBP-COQ Prof© questionnaire. Bivariate and multiple lineal regression analyses were carried out.

**Results:**

The mean score for the EBP competency of the PC nurses was 131.5 (standard deviation [SD] 17.0), according to dimensions: attitude 36.8 (SD 3.6); knowledge 38.2 (SD 8.9); skills 23.0 (SD 3.5); and utilization 33.3 (SD 6.1). The number of articles read in the last month has showed the most influence on all the EBP-COQ Prof© dimensions, followed by EBP training (more than 150 h) and nursing students mentoring. The education level (master, specialist and doctorate) is associated with knowledge and skills dimensions, meanwhile belonging to a BPSO® center is associated with the EBP utilization.

**Conclusions:**

These findings can guide PC service managers to plan strategies that improve the EBP competency level of the nurses, aimed mainly at achieving real application in clinical practice. However, it is necessary to consider the possible impact of selection bias on the results.

## Introducción

En la actualidad, el envejecimiento de la población, la elevada morbilidad de las personas mayores^1^ y el entorno profesional cambiante al que se enfrentan las enfermeras en el ámbito de la salud pública y comunitaria precisan que se garanticen unos cuidados de enfermería seguros y de calidad^2^. Las enfermeras representan casi el 50% de los profesionales sanitarios a nivel mundial y, especialmente en el ámbito de la Atención Primaria (AP), desempeñan un papel fundamental en la promoción de la salud y la prevención de enfermedades^3^. Prestan sus cuidados en la primera línea de la atención sanitaria, por lo que tienen una posición clave para cuestionar la práctica y utilizar los resultados de investigación en la toma de decisiones^4^.

La incorporación de la Práctica Basada en la Evidencia (PBE) en los servicios de salud mejora la calidad de la atención sanitaria, aumenta la seguridad del paciente, reduce costes, mejora los resultados en salud y aumenta la satisfacción laboral de las enfermeras^5^, ^6^. La Organización Mundial de la Salud considera la PBE un área de actuación prioritaria para aumentar la contribución de las enfermeras a la salud de la ciudadanía^7^. Además, las principales organizaciones de profesionales de la salud, incluidas las de AP, consideran la competencia en PBE como un estándar profesional a tener en cuenta por los servicios sanitarios^2^, ^8^.

A nivel internacional existe interés por identificar el nivel de competencia en PBE de las enfermeras^9^, ^10^, siendo más frecuentes los estudios desarrollados en entornos hospitalarios^11^. Las conclusiones globales señalan que gran parte de las enfermeras no se sienten competentes para aplicar la PBE, independientemente del entorno de trabajo (atención hospitalaria o primaria) donde facilitan los cuidados^11^, y preferentemente toman sus decisiones según las opiniones y experiencias previas de sus compañeras de trabajo^12^.

Recientemente, una revisión sistemática evaluó los conocimientos, actitudes, barreras, facilitadores e implantación de la PBE en enfermeras comunitarias, incluyendo estudios de 9 países distintos, y sus resultados muestran que las enfermeras tienen interés por la PBE y la consideran útil para mejorar la calidad de la atención sanitaria^13^. También muestran la existencia de numerosas barreras que limitan su aplicación en la práctica clínica, principalmente la necesidad de tiempo y recursos, la falta de conocimientos y habilidades, así como de estímulo y soporte adecuado por parte de los servicios de salud^13^. Por otra parte, se han descrito aspectos facilitadores del uso de la PBE, como la edad y el nivel de formación, observándose que las enfermeras más jóvenes recurren menos a la intuición o experiencia, y las enfermeras expertas y con un nivel de educación superior tienen más habilidades en la síntesis de información científica^14^. También se han observado como factores mediadores en la competencia en PBE las características de las evidencias y las habilidades de las enfermeras en lectura de artículos científicos^13^, la existencia de enfermeras de práctica avanzada y especialistas en AP como mentoras y promotoras de la PBE y diseminadoras del conocimiento^15^, así como la importancia de un contexto de trabajo favorable, en el que los gestores puedan facilitar tiempo y herramientas a las enfermeras comunitarias para utilizar la PBE de forma adecuada^16^.

Los estudios españoles realizados a nivel regional presentan resultados similares a la literatura internacional, y encuentran barreras para el uso de la PBE como la falta de tiempo y la escasez de conocimientos y habilidades^17^, ^18^, ^19^, ^20^. Sin embargo, carecemos de datos actualizados en España, por lo que el objetivo de este estudio fue conocer el nivel de competencia en PBE de enfermeras en AP y analizar los factores explicativos que pueden influir en esta competencia en el contexto de la AP española.

## Métodos

### Diseño y sujetos de estudio

Estudio transversal de carácter nacional desarrollado en España durante el año 2020, estudio #Evidencer^21^. El estudio #Evidencer seleccionó a través de un muestreo no probabilístico estratificado por comunidades autónomas a las enfermeras que estuvieran en activo en el Sistema Nacional de Salud en España con una experiencia profesional mínima de un año, obteniendo una muestra de 2.982 enfermeras de toda España. Para realizar el presente estudio se ha seleccionado la submuestra de 780 enfermeras de AP.

### Variables, instrumento y proceso de recogida de datos

La recogida de datos se realizó mediante un cuestionario online realizado a través de redes sociales y contactando por correo electrónico y telefónico con asociaciones de enfermería y colegios profesionales, durante los meses de enero a marzo de 2020.

El cuestionario, de 4 páginas, de acceso abierto y no revisable posteriormente, se realizó a través de Formularios de Google. No existió control sobre si una persona contestó más de una vez el formulario. El tiempo de cumplimentación era aproximadamente de 15 min. Incluía las siguientes variables:

- Sociodemográficas: edad, sexo y carga familiar.
- Profesionales: año de finalización de los estudios, años de experiencia profesional, años de trabajo en AP, entorno de trabajo (rural o urbano), situación laboral, tipo de jornada laboral, nivel educativo máximo alcanzado, formación en PBE, número de artículos leídos en el último mes, tutorización de estudiantes de Enfermería, trabajar en un centro Best Practice Spotlight Organization (BPSO®), centros sanitarios que participan en el programa internacional de implantación de guías de práctica clínica desarrollado por la Registered Nurses’ Association of Ontario^22^.
- Relacionadas con el acceso a información científica: utilización de Internet y redes sociales, frecuencia de uso, utilización de Twitter, blogs de salud, lugar de acceso más frecuente a Internet, acceso a Internet en el centro de trabajo.
- Variable de resultado: competencia en PBE evaluada mediante el cuestionario Competencia en Práctica Basada en la Evidencia en profesionales (EBP-COQ Prof©), validado en el contexto español, con una validez y fiabilidad adecuadas^23^. El cuestionario se desarrolló a partir del marco competencial en PBE para enfermeras de cuidados generales propuesto por Melnyk et al.^24^, y la definición operativa de competencia utilizada es «la capacidad de la enfermera para integrar las habilidades cognitivas, afectivas y psicomotoras en la prestación de cuidados de enfermería»^25^. Esta definición incluye: conducta potencial (actitudes, conocimientos y habilidades) y conducta real (utilización de la PBE en el contexto clínico)^26^. El cuestionario consta de 35 ítems que se responden con una escala Likert de 1 a 5, organizado en 4 dimensiones: actitud (8 ítems, rango de puntuación 8-40), conocimientos (11 ítems, rango 11-55), habilidades (6 ítems, rango 6-30) y utilización (10 ítems, rango 10-50). La puntuación global del nivel de competencia en PBE tiene un rango entre 35 y 175 puntos (a mayor puntuación, mayor competencia).

### Análisis de datos

Se realizó un análisis univariante, descriptivo, con medidas de tendencia central para las variables sociodemográficas cuantitativas y las dimensiones del EBP-COQ Prof©, y se calcularon las frecuencias absolutas y los porcentajes para las cualitativas. Se realizó un análisis bivariante entre las puntuaciones de las dimensiones y la puntuación total del EBP-COQ Prof©, y las variables sociodemográficas y profesionales, mediante pruebas estadísticas de ANOVA de un factor y la correlación de Pearson. Se realizaron modelos multivariantes (regresión lineal múltiple) para conocer la influencia de las variables analizadas sobre las dimensiones actitud, conocimientos, habilidades, utilización y puntuación total del cuestionario EBP-COQ Prof©. Se empleó el método secuencial por pasos hacia adelante (forward) con los criterios probabilidad de F para entrar ≤ 0,05 y salir  > 0,10. En el análisis estadístico se consideró un nivel de significación del 5% (p ≤ 0,05). Los análisis se realizaron con el programa SPSS® 26.0.

### Consideraciones éticas

El estudio fue aprobado por el Comité de Ética de la Universidad de Murcia (ID: 2540/2019). Las enfermeras participaron de forma voluntaria. No se requirió ningún dato que pudiera identificar a las participantes y se garantizó la confidencialidad. Las participantes disponían de una hoja con la información sobre la participación en el proyecto (incluía dónde y por cuánto tiempo se almacenaban los datos, quién era el investigador principal y el objetivo del estudio), y para acceder al cuestionario debían previamente aceptar el consentimiento informado.

**Figure:**
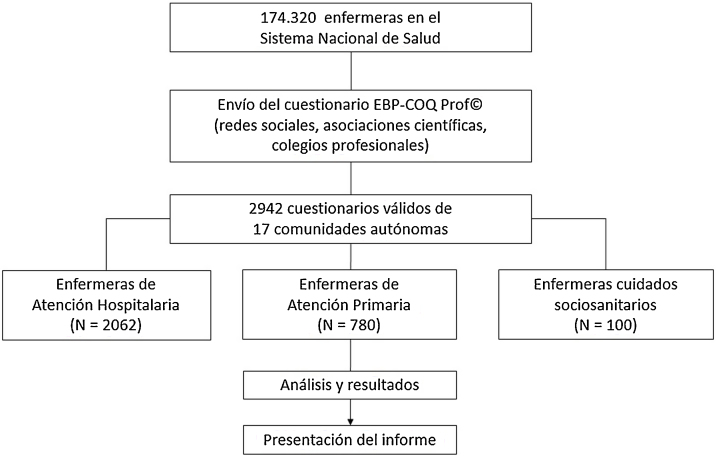
**Esquema general del estudio**.

## Resultados

La página web donde se incluía el acceso al cuestionario recibió 18.897 visitas. Se recogieron 2.942 cuestionarios de enfermeras de todo el territorio nacional. El cálculo aproximado de la tasa de participación es del 15,6%, aunque puede estar sobreestimado por las condiciones de acceso al cuestionario. Concretamente, para este estudio la muestra analizada fue de 780 enfermeras que trabajan en el ámbito de AP. Seiscientas participantes (76,9%) eran mujeres, con una edad media de 43,6 años (desviación típica [DT] 10,1), experiencia laboral media global de 19,8 años (DT 10,5) y 12,8 años (DT 10,1) de experiencia en AP. Más de trescientas (42,3%) trabajaban en un entorno urbano y 445 participantes (57,1%) tenía un contrato fijo. Respecto de la formación, 429 enfermeras (55%) tenía formación posgrado y la mayoría habían realizado formación en PBE y accedían a Internet para consultar información científica (tabla 1).

**Tabla 1:** Variables sociodemográficas y profesionales de la muestra

	M	DT
*Edad (años)*	43,6	10,1
*Años desde que finalizó los estudios de Enfermería*	21,9	10,5
*Experiencia profesional (años)*	19,8	10,5
*Experiencia profesional en Atención Primaria (años)*	12,8	10,1
	n	%
*Experiencia profesional (años)*		
10 o menos	184	23,6
11-22	257	32,9
23-33	263	33,7
34 o más	76	9,7

*Sexo*		
Hombre	180	23,1
Mujer	600	76,9

*Carga familiar*		
Sí	498	63,8
No	282	36,2

*Nivel educativo*		
Diplomatura/Grado	351	45,0
Máster	272	34,9
Especialidad	109	14,0
Doctorado	48	6,2

*Situación laboral*		
Eventual	179	22,9
Interino	156	20,0
Fijo	445	57,1

*Dedicación laboral*		
Tiempo total	745	95,5
Tiempo parcial	35	4,5

*Entorno del centro de trabajo*		
Urbano (> 50.000 habitantes)	330	42,3
Suburbano (entre 10.000 y 50.000 habitantes)	247	31,7
Rural (< 10.000 habitantes)	203	26,0

*Formación en Práctica Basada en la Evidencia (horas)*		
Ninguna	110	14,1
< 40	193	24,7
40-150	253	32,4
> 150	224	28,7

*Número de artículos científicos leídos en el último mes*		
0	154	19,7
Entre uno y 3	339	43,5
> 3	287	36,8

*Trabaja en un centro BPSO®*		
Sí	100	12,8
No	528	67,7

*Tutorización de alumnos/as de Enfermería*		
Sí	395	50,6
No	385	49,4

*Uso de Internet y otras herramientas digitales para acceder a información científica*		
Sí	648	83,1
No	132	16,9

*Uso de Twitter para acceder a información científica*		
Sí	337	43,2
No	443	56,8

*Uso de blogs de salud para acceder a información científica*		
Sí	539	69,1
No	241	30,9

*Acceso a Internet en el trabajo*		
Sí	719	92,2
No	61	7,8

*Lugar de acceso a Internet más frecuente para consultar información científica*		
Casa	590	75,6
Trabajo	190	24,4

*Frecuencia de uso de Internet y otras herramientas digitales para acceder a información científica*		
Nunca	82	10,5
Ocasionalmente	221	28,3
Mensualmente	62	7,9
Semanalmente	170	21,8
Diariamente	245	31,4

*Comunidad Autónoma*		
Andalucía	284	36,4
Aragón	6	0,8
Asturias	22	2,8
Cantabria	16	2,1
Castilla-La Mancha	27	3,5
Castilla y León	80	10,3
Cataluña	56	7,2
Comunidad Valenciana	27	3,5
Extremadura	9	1,2
Galicia	22	2,8
Islas Baleares	21	2,7
Islas Canarias	20	2,6
La Rioja	9	1,2
Madrid	58	7,4
Murcia	79	10,1
Navarra	9	1,2
País Vasco	35	4,5

BPSO®: Best Practice Spotlight Organization; DT: desviación típica; M: media.

La puntuación media en el nivel de competencia en PBE de las enfermeras de AP españolas fue de 131,5 (DT 17,0). Los resultados por cada una de las dimensiones del EBP-COQ Prof© mostraron una puntuación media de 36,8 (DT 3,6) en actitud, 38,2 (DT 8,9) en conocimientos, 23,0 (DT 3,5) en habilidades y 33,3 (DT 6,1) en utilización de la PBE.

**Figura 1:**
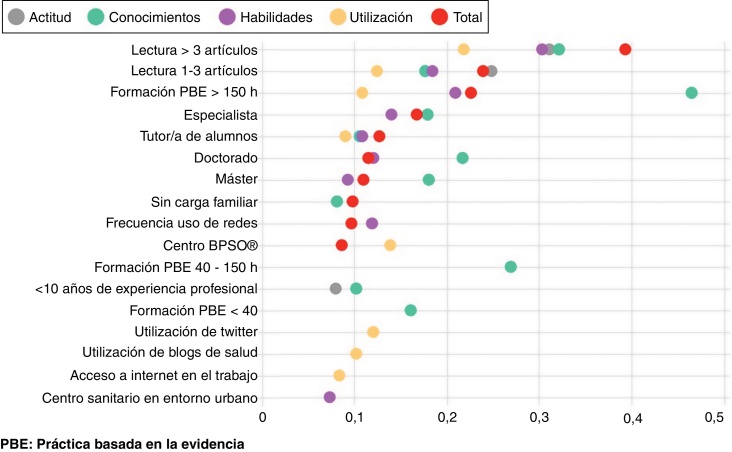
Puntuaciones estandarizadas del efecto de las variables por dimensiones y total del EBP-COQ Prof®.

Los resultados de los análisis bivariados muestran una asociación estadísticamente significativa (p < 0,05) entre un número importante de variables sociodemográficas, profesionales y de acceso a la información científica y las dimensiones y la puntuación total del EBP-COQ Prof© (Tabla 2, Tabla 3, Tabla 4). El análisis multivariado revela las variables definitivas retenidas en los modelos ajustados y la magnitud del efecto asociado con las 4 dimensiones y la puntuación total del EBP-COQ Prof© (tabla 5). El número de artículos leídos en el último mes es la variable que más influye en todas las dimensiones del EBP-COQ Prof©, seguida de la formación en PBE (más de 150 h) y la tutorización de alumnos de Enfermería, que se asocian con la mejora de los conocimientos, las habilidades y la utilización de la PBE. El nivel educativo (máster, especialista y doctorado) se asocia con las dimensiones conocimientos y habilidades, mientras que trabajar en un centro BPSO® se asocia con la utilización de la PBE. En la figura 1 se presenta una visión global de las puntuaciones estandarizadas del efecto de las variables explicativas por dimensiones y el total del EBP-COQ Prof©.

**Tabla 2:** Resultados bivariados del EBP-COQ Prof© comparados con variables sociodemográficas de las enfermeras de Atención Primaria

		Actitud		Conocimientos		Habilidades		Utilización		Total	
Características	N	r	p	r	p	r	p	r	p	r	p
*Edad*	780	−0,099	0,006	−0,172	< 0,001	−0,108	0,002	0,005	0,898	−0,133	< 0,001
*Años finalización estudios*	780	−0,103	0,004	−0,172	< 0,001	−0,099	0,005	0,015	0,685	−0,128	< 0,001
*Años experiencia profesional*	780	−0,097	0,007	−0,154	< 0,001	−0,083	0,021	0,018	0,613	−0,113	0,002
*Años Atención Primaria*	780	−0,021	0,551	−0,051	0,153	−0,029	0,416	0,043	0,231	−0,022	0,537

	N (%)	M (DT)	p	M (DT)	p	M (DT)	p	M (DT)	p	M (DT)	p
*Sexo*			0,400		0,008		0,039		0,493		0,161
Hombre	180 (23,1)	36,6 (3,5)		39,7 (8,7)		23,5 (3,5)		33,1 (5,8)		133,0 (16,9)	
Mujer	600 (76,9)	36,9 (3,7)		37,7 (9,0)		22,9 (3,5)		33,4 (6,2)		131,0 (17,0)	
*Carga familiar*			0,018		< 0,001		0,003		0,410		< 0,001
Sí	498 (63,8)	36,6 (3,5)		36,9 (9,1)		22,7 (3,6)		33,2 (6,1)		129,6 (17,3)	
No	282 (36,2)	37,2 (3,8)		40,4 (8,2)		23,5 (3,3)		33,6 (6,1)		134,9 (15,9)	
*Nivel educativo*			0,001		< 0,001		< 0,001		0,422		< 0,001
a. Diplomado/Grado	351 (45,0)	36,4 (3,6)^c^		34,3 (8,5)^b,c,d^		22,0 (3,5)^b,c,d^		33,1 (6,0)		126,0 (16,1)^b,c,d^	
b. Máster	272 (34,9)	36,8 (3,9)^c^		39,7 (8,2)^a,c,d^		23,4 (3,3)^a,d^		33,3 (6,1)		133,4 (16,5)^a,c,d^	
c. Enfermera Especialista	109 (14,0)	37,9 (2,4)^a,b^		42,6 (7,1)^a,b,d^		24,2 (2,9)^a,d^		34,2 (6,0)		139,0 (14,6)^a,b,d^	
d. Doctorado	48 (6,2)	37,3 (4,3)		47,2 (5,9)^a,b,c^		25,7 (2,9)^a,b,c^		33,6 (7,4)		144,0 (16,8)^a,b^	
*Formación en PBE (horas)*			0,001		< 0,001		< 0,001		< 0,001		< 0,001
a. Ninguna	110 (14,1)	35,8 (4,4)^d^		30,2 (8,7)^b,c,d^		21,1 (3,7)^c,d^		31,9 (6,2)^d^		119,2 (16,7)^b,c,d^	
b. <40	193 (24,7)	36,4 (3,8)^d^		35,1 (8,4)^a,c,d^		22,0 (3,5)^c,d^		32,6 (6,5)^d^		126,3 (16,5)^a,c,d^	
c. 40-150	253 (32,4)	37,0 (3,3)		39,1 (7,0)^a,b,d^		23,1 (3,1)^a,b,d^		33,2 (5,3)^d^		132,5 (13,4)^a,b,d^	
d. >150	224 (28,7)	37,3 (3,3)^a,b^		43,6 (7,5)^a,b,c^		24,8 (3,0)^a,b,c^		34,8 (6,3)^a,b,c^		140,7 (15,8)^a,b,c^	
*Artículos leídos último mes*			< 0,001		< 0,001		< 0,001		< 0,001		< 0,001
a. 0	154 (19,7)	35,0 (5,1)^b,c^		31,4 (8,2)^b,c^		20,8 (3,5)^b,c^		31,1 (6,5)^b,c^		118,5 (16,3)^b,c^	
b. Entre 1 y 3	339 (43,5)	37,0 (3,0)^a^		37,3 (7,9)^a,d^		22,8 (3,3)^a,c^		33,1 (5,8)^a,b^		130,5 (14,5)^a,c^	
c. >3	287 (36,8)	37,5 (3,0)^a^		42,8 (7,8)^a,b^		24,5 (3,1)^a,b^		34,8 (5,9)^a,b^		139,7 (15,4)^a,b^	

DT: desviación típica; M: media; PBE: Práctica Basada en la Evidencia; r: correlación de Pearson.

Las letras en superíndice indican, según las pruebas *post hoc* Games-Howell del análisis de la varianza, entre qué categorías de la variable analizada hay asociación estadística p < 0,05.

**Tabla 3:** Resultados bivariados del EBP-COQ Prof© comparados con variables laborales de las enfermeras de Atención Primaria

		Actitud		Conocimientos		Habilidades		Utilización		Total	
Características	N (%)	M (DT)	p	M (DT)	p	M (DT)	p	M (DT)	p	M (DT)	p
*Situación laboral*			0,163		0,107		0,656		0,230		0,358
a. Eventual	179 (22,9)	36,8 (3,4)		39,2 (8,9)		23,1 (3,1)		32,7 (5,7)		131,9 (16,6)	
b. Interino	156 (20,0)	37,3 (3,7)		38,6 (8,0)		23,2 (3,5)		33,7 (6,2)		133,0 (16,1)	
c. Fijo	445 (57,1)	36,6 (3,7)		37,6 (9,3)		22,9 (3,7)		33,5 (6,2)		130,8 (17,4)	
*Dedicación laboral*			0,337		0,868		0,340		0,938		0,602
Tiempo total	745 (95,5)	36,8 (3,6)		38,2 (8,9)		23,0 (3,5)		33,3 (6,1)		131,5 (17,0)	
Tiempo parcial	35 (4,5)	36,2 (3,4)		37,9 (9,3)		22,5 (3,6)		33,3 (5,9)		133,0 (17,5)	
*Trabaja en un centro BPSO®*			0,485		0,042		0,014		< 0,001		0,002
Sí	100 (12,8)	36,9 (4,3)		40,7 (8,0)		24,1 (3,0)		35,7 (6,3)		137,5 (16,1)	
No	528 (67,7)	37,1 (3,4)		38,8 (8,8)		23,2 (3,4)		32,7 (6,1)		131,9 (16,8)	
*Tutorización alumnos/as de Enfermería*			0,592		< 0,001		< 0,001		< 0,001		< 0,001
Sí	395 (50,6)	36,9 (3,8)		39,6 (8,6)		23,7 (3,4)		34,4 (6,0)		134,7 (16,6)	
No	385 (49,4)	36,7 (3,5)		36,7 (9,1)		22,4 (3,5)		32,2 (6,0)		128,1 (16,9)	
*Entorno del centro de trabajo*			0,340		0,050		0,004		0,001		0,006
a. Urbano (> 50.000 habitantes)	330 (42,3)	36,9 (3,3)		38,9 (8,9)^c^		23,5 (3,3)^b,c^		34,1 (6,3)^c^		133,5 (17,0)^c^	
b. Suburbano (entre 10.000-50.000 habitantes)	247 (31,7)	36,5 (3,7)		38,2 (8,9)		22,8 (3,5)^a^		33,4 (5,9)		131,0 (16,5)	
c. Rural (< 10.000 habitantes)	203 (26,0)	37,0 (4,0)		37,0 (9,0)^a^		22,5 (3,7)^a^		32,1 (6,0)^a^		128,7 (17,3)^a^	

BPSO®: Best Practice Spotlight Organization; DT: desviación típica; M: media.

Las letras en superíndice indican, según las pruebas *post hoc* Games-Howell del análisis de la varianza, entre qué categorías de la variable analizada hay asociación estadística p < 0,05.

**Tabla 4:** Resultados bivariados del EBP-COQ Prof© comparados con variables relacionadas con el acceso a información científica a través de Internet

		Actitud		Conocimientos		Habilidades		Utilización		Total	
Características	N (%)	M (DT)	p	M (DT)	p	M (DT)	p	M (DT)	p	M (DT)	p
*Uso de Internet y herramientas digitales acceso información científica*			0,090		0,062		0,021		0,035		0,009
Sí	648 (83,1)	39,9 (3,5)		38,4 (8,7)		23,2 (3,4)		33,6 (6,0)		132,2 (16,3)	
No	132 (16,9)	36,3 (3,9)		36,8 (10,0)		22,4 (3,7)		32,3 (6,4)		128,0 (19,7)	
*Frecuencia de uso de Internet y otras herramientas digitales*			0,001		< 0,001		< 0,001		< 0,001		< 0,001
a. Nunca	82 (10,5)	36,2 (4,4)		37,3 (10,5)^e^		22,6 (4,0)^e^		32,4 (6,3)		128,6 (20,7)^e^	
b. Ocasionalmente	221 (28,3)	36,2 (3,6)^e^		35,3 (8,6)^d,e^		21,6 (3,7)^c,d,e^		32,0 (6,4)^d,e^		125,3(16,1)^d,e^	
c. Mensualmente	62 (7,9)	37,1 (2,9)		36,9 (7,9)^e^		23,0 (2,9)^b^		33,3 (5,7)		130,5 (13,5)^e^	
d. Semanalmente	170 (21,8)	36,6 (4,0)		38,6 (8,2)^b,e^		23,5 (3,0)^b^		34,0 (5,8)^b^		132,8 (16,0)^b,e^	
e. Diariamente	245 (31,4)	37,5 (3,1)^b^		41,1 (8,5)^a,b,c,d^		24,1 (3,1)^a,b^		34,5 (5,9)^b^		137,4 (15,8)^a,b,c,d^	
*Lugar acceso Internet más frecuente consulta información científica*			0,865		0,778		0,357		0,302		0,498
Casa	590 (75,6)	36,8 (3,7)		38,1 (8,8)		23,0 (3,5)		33,2 (6,0)		131,2 (16,6)	
Trabajo	190 (24,4)	36,8 (3,5)		38,3 (9,4)		23,2 (3,6)		33,7 (6,3)		132,2 (18,3)	
*Uso de Twitter para acceder a información científica*			0,001		< 0,001		< 0,001		0,001		< 0,001
Sí	337 (43,2)	37,3 (3,5)		40,5 (8,3)		23,7 (3,2)		34,2 (5,6)		135,8 (15,3)	
No	443 (56,8)	36,4 (3,7)		36,4 (9,0)		22,5 (3,6)		32,7 (6,4)		128,2 (17,4)	
*Uso de blogs de salud para acceder a información científica*			0,003		< 0,001		< 0,001		< 0,001		< 0,001
Sí	539 (69,1)	37,1 (3,6)		39,3 (8,7)		23,4 (3,2)		34,0 (6,0)		133,3 (16,1)	
No	241 (30,9)	36,2 (3,7)		36,3 (9,2)		22,3 (3,9)		31,9 (6,1)		126,8 (18,0)	
*Acceso a Internet en el trabajo*			0,668		0,851		0,338		0,845		0,950
Sí	719 (92,2)	36,8 (3,7)		38,2 (8,9)		23,1 (3,5)		33,3 (6,2)		131,5 (17,1)	
No	61 (7,8)	37,0 (2,9)		38,4 (9,1)		22,6 (3,2)		33,5 (5,6)		131,6 (15,6)	

DT: desviación típica; M: media.

Las letras en superíndice indican, según las pruebas *post hoc* Games-Howell del análisis de la varianza, entre qué categorías de la variable analizada hay asociación estadística p < 0,05.

**Tabla 5:** Modelos de regresión lineal múltiple por dimensiones y en la competencia total según el EBP-COQ Prof©

Variables dependientes	Variables independientes	Coeficientes no estandarizado	Intervalo de confianza al 95% para B		Coeficientes estandarizados	p
		B	Límite inferior	Límite superior	Beta	
Actitud	Número de artículos leídos (más de 3 en el último mes)	2,320	1,521	3,119	0,311	< 0,001
	Número de artículos leídos (entre 1 y 3 en el último mes)	1,831	1,042	2,621	0,249	< 0,001
	< 10 años de experiencia professional	0,655	0,029	1,281	0,080	0,040
Conocimientos	Formación en PBE (más de 150 h)	8,780	6,893	10,667	0,465	< 0,001
	Número de artículos leídos (más de 3 en el último mes)	5,754	4,047	7,460	0,322	< 0,001
	Número de artículos leídos (entre 1 y 3 en el último mes)	3,134	1,570	4,698	0,177	< 0,001
	Doctorado	7,300	5,058	9,543	0,217	< 0,001
	Especialista	4,240	2,457	6,022	0,179	< 0,001
	Máster	3,226	1,995	4,456	0,180	< 0,001
	Formación en PBE (entre 40 y 150 h)	5,083	3,256	6,909	0,269	< 0,001
	Formación en PBE (menos de 40 h)	3,295	1,481	5,108	0,161	< 0,001
	< 10 años de experiencia profesional	2,012	0,493	3,532	0,102	0,010
	Tutor/a de alumnos/as de Enfermería	1,848	0,711	2,984	0,106	0,001
	No tener carga familiar	1,444	0,170	2,719	0,081	0,026
Habilidades	Formación en PBE (más de 150 h)	1,529	0,989	2,069	0,210	< 0,001
	Número de artículos leídos (más de 3 en el último mes)	2,089	1,343	2,836	0,303	< 0,001
	Número de artículos leídos (entre 1 y 3 en el último mes)	1,264	0,581	1,947	0,185	< 0,001
	Frecuencia uso de Internet y redes sociales	0,279	0,107	0,451	0,119	0,001
	Tutor/a de alumnos	0,738	0,251	1,225	0,109	0,003
	Especialidad	1,277	0,562	1,992	0,140	< 0,001
	Doctorado	1,559	0,571	2,547	0,120	0,002
	Máster	0,648	0,105	1,191	0,093	0,019
	Centro sanitario en entorno urbano	0,495	0,015	0,975	0,073	0,043
Utilización	Número de artículos leídos (más de 3 en el último mes)	2,803	1,383	4,223	0,219	< 0,001
	Centro BPSO®	2,386	1,112	3,659	0,139	< 0,001
	Utilización de Twitter para acceso a información científica	1,498	0,529	2,468	0,120	0,003
	Formación en PBE (más de 150 h)	1,479	0,429	2,530	0,109	0,006
	Tutor/a de alumnos/as de Enfermería	1,136	0,201	2,071	0,091	0,017
	Utilización de blogs de salud para acceso a información científica	1,444	0,374	2,515	0,103	0,008
	Número de artículos leídos (entre 1 y 3 en el último mes)	1,568	0,235	2,900	0,124	0,021
	Acceso con frecuencia a Internet en el trabajo	1,201	0,124	2,279	0,084	0,029
Competencia total	Número de artículos leídos (más de 3 en el último mes)	13,562	10,056	17,068	0,393	< 0,001
	Formación en PBE (más de 150 h)	8,234	5,702	10,767	0,226	< 0,001
	Número de artículos leídos (entre 1 y 3 en el último mes)	8,201	4,995	11,406	0,240	< 0,001
	Especialista	7,641	4,123	11,160	0,168	< 0,001
	Tutor/a de alumnos/as de Enfermería	4,281	1,969	6,592	0,127	< 0,001
	Frecuencia uso de Internet y redes sociales	1,140	0,334	1,945	0,097	0,006
	No tener carga familiar	3,364	0,951	5,777	0,098	0,006
	Centro BPSO®	4,027	0,994	7,060	0,087	0,009
	Doctorado	7,512	2,873	12,151	0,116	0,002
	Máster	3,817	1,261	6,373	0,110	0,003

BPSO®: Best Practice Spotlight Organization; PBE: Práctica Basada en la Evidencia.

Actitud: modelo 3, R^2^ = 0,05; Conocimientos: modelo 11, R^2^ = 0,41; Habilidades: modelo 9, R^2^ = 0,23; Utilización: modelo 8, R^2^ = 0,13; Competencia total: modelo 10, R^2^ = 0,32.

## Discusión

El presente estudio describe por primera vez el nivel de competencia en PBE en una muestra de enfermeras de AP españolas mediante el EBP-COQ Prof©^23^. Este es un instrumento apropiado para recoger las dos vertientes que incluye la definición operativa de competencia: la conducta potencial y la conducta real, que depende de las circunstancias y el entorno^26^. Además, se han estudiado los factores y la magnitud de su asociación con las dimensiones del cuestionario.

El perfil sociodemográfico y profesional de las participantes es similar al de las enfermeras que trabajan en centros de AP del Sistema Nacional de Salud^27^. Sin embargo, a pesar de las funciones, del trabajo más colaborativo y del mayor grado de autonomía de estas profesionales^19^, presentan un nivel de competencia en PBE similar al descrito en estudios nacionales e internacionales realizados en el entorno hospitalario^11^ y de AP^12^, ^13^, mostrando actitudes favorables hacia la PBE, aunque con habilidades y conocimientos moderados, y niveles bajos de utilización de la PBE. Sin duda, en el ámbito de la AP se requiere hacer un importante esfuerzo dirigido a aumentar los conocimientos y habilidades de las enfermeras, una de las principales barreras para la PBE^13^, y sobre todo a mejorar la utilización real de la PBE en la práctica clínica^7^. Los factores explicativos observados en nuestros hallazgos señalan algunas claves para orientar dichos esfuerzos.

La formación continuada en PBE y el nivel educativo de las enfermeras tienen una importante influencia en la competencia en PBE, aunque con matices. La formación continuada en PBE mejora los conocimientos y habilidades, sin embargo, se asocia con la utilización de la PBE cuando la formación supera las 150 h. En este sentido, se ha señalado que enfermeras que han recibido formación breve en PBE mejoraron sus conocimientos/habilidades, no así su práctica^28^. Por otra parte, los resultados muestran que tener un máster, un doctorado o una especialidad explica mayores conocimientos y habilidades en PBE, pero no se asocia con una mayor utilización. Posiblemente, como muestran otros estudios, se precisa contar con un contexto y organización del trabajo favorables para implementar dicho conocimiento^11^, ^29^. En otros países se ha implantado con éxito la figura de la enfermera mentora en PBE, rol que en la AP en España podrían desempeñar las enfermeras especialistas comunitarias, quienes con el soporte adecuado, pueden liderar procesos de implantación y ser motores de cambio hacia un cultura organizativa basada en la PBE^11^, ^14^, ^15^.

Otro de los aspectos más significativos de nuestros hallazgos se relaciona con los factores asociados con una mayor utilización de la PBE en la práctica clínica. Concretamente, el número de artículos leídos en el último mes es la variable que más influye en la competencia en PBE, y la que presenta un efecto más fuerte en la utilización. Otros estudios señalan una débil influencia sobre la competencia en PBE^17^. En este sentido, la incorporación de iniciativas como los Journal Clubs, que ya han mostrado resultados positivos^30^, podría ser de interés en AP. Por otro lado, la aplicación actual en España del programa de implantación de guías de práctica clínica denominado BPSO® nos ha brindado un escenario único, que evidencia que aplicar este tipo de programas de implantación puede ser una estrategia efectiva para mejorar la utilización de la PBE^31^. Como se observa en la figura 1, este programa tiene un efecto muy focalizado en la dimensión utilización de la PBE. Esto nos hace pensar, en términos de Sackett, que el programa BPSO® promueve el uso de la PBE según el modo «repetir»^32^, en el que las enfermeras replican en la práctica clínica las recomendaciones para la implantación indicadas por las líderes de las guías. Asimismo, se ha observado como factor explicativo importante la tutorización de alumnos de Enfermería, también asociado a los conocimientos y habilidades en PBE. Como plantean algunos estudios, la interacción entre estudiantes y enfermeras puede ayudar a integrar mejor la PBE en ambas poblaciones^33^. Finalmente, el uso de redes sociales y blogs de salud para la consulta de información científica y el acceso frecuente a Internet en el trabajo también se ha asociado a la utilización de la PBE, lo que respalda que se facilite este modo de acceso a las evidencias en el entorno laboral.

Por último, hay que señalar que en la planificación de actividades en AP para el fomento de la PBE se deberían establecer estrategias adaptadas a distintas características de los profesionales y su entorno: con carga familiar, mayor experiencia laboral y/o que trabajen en centros de salud rurales.

### Limitaciones

Las limitaciones se derivan de la selección no probabilística de la muestra y la recogida de datos online, lo que puede limitar la representatividad y extrapolación de los resultados. Es posible que una persona rellenase el cuestionario más de una vez. Este sesgo se intentó reducir contactando con las enfermeras a través de diferentes vías (correo electrónico, redes sociales, asociaciones profesionales) y estratificando por las diferentes comunidades autónomas. A pesar del carácter aproximado del cálculo de la tasa de participación, la tasa es baja y existe una participación desigual por parte de las comunidades autónomas, también influido por el número de enfermeras existentes en cada una. Asimismo, los factores estudiados son limitados y se requieren futuras investigaciones que busquen nuevas variables explicativas asociadas principalmente a la utilización de la PBE.

## Conclusiones

Las enfermeras de AP estudiadas tienen una actitud positiva, conocimientos y habilidades moderadas y niveles bajos en la dimensión utilización de la PBE. Los factores que han mostrado mayor influencia en los conocimientos, las habilidades y la utilización de la PBE son la lectura de artículos científicos, la formación continuada en PBE mayor de 150 h y ser tutor de alumnos en las prácticas clínicas. Las enfermeras con formación de posgrado (máster, especialidad y doctorado) muestran una relación positiva creciente con los conocimientos y habilidades que no se observa con el uso de la PBE. Por otra parte, se muestra una asociación significativa de la utilización de la PBE y trabajar en un centro BPSO®, la utilización de redes sociales y el acceso frecuente a Internet en el trabajo. Estos hallazgos pueden orientar a gestores en los servicios de AP a planificar estrategias que mejoren el nivel de competencia en PBE de las enfermeras, ampliando los esfuerzos (que tradicionalmente se han dirigido a mejorar la actitud, las habilidades y los conocimientos en PBE) a lograr una utilización real de la misma. No obstante, se hace necesario considerar el posible impacto del sesgo de selección en los resultados.

**Lo conocido sobre el tema**

• Las principales organizaciones de profesionales de la salud consideran la competencia en PBE un estándar profesional a tener en cuenta por los servicios de salud.
• Gran parte de las enfermeras no se sienten competentes para aplicar la PBE independientemente del entorno de trabajo. Entre las barreras destacan: necesidad de tiempo y recursos, falta de conocimientos y habilidades, y apoyo inadecuado de los servicios de salud.
• Los estudios españoles realizados a nivel regional presentan resultados similares a la literatura internacional, aunque carecemos de datos actualizados en España.

**Qué aporta este estudio**

• Las enfermeras de AP estudiadas tienen una actitud positiva, conocimientos y habilidades moderadas y niveles bajos en la dimensión utilización de la PBE.
• La lectura de artículos científicos, formación continuada en PBE mayor de 150 h y ser tutor/a de estudiantes de Enfermería son los factores con más influencia en los conocimientos, habilidades y utilización de la PBE. Existe una asociación significativa entre la utilización de la PBE y trabajar en un centro BPSO®, la utilización de redes sociales y el acceso frecuente a Internet en el trabajo.
• Los servicios de AP deberían aspirar a realizar estrategias cuyo objetivo final sea una aplicación real de la PBE.

## Financiación

Esta investigación ha sido financiada por la Agencia Estatal de Investigación: PID2019-106545GA-I00/AEI/10.13039/501100011033.

## Conflicto de intereses

Los autores declaran no tener ningún conflicto de intereses.
